# Short-Term Responses of Soil Respiration and C-Cycle Enzyme Activities to Additions of Biochar and Urea in a Calcareous Soil

**DOI:** 10.1371/journal.pone.0161694

**Published:** 2016-09-02

**Authors:** Dali Song, Xiangyin Xi, Shaomin Huang, Guoqing Liang, Jingwen Sun, Wei Zhou, Xiubin Wang

**Affiliations:** 1 Institute of Agricultural Resource and Regional Planning, Chinese Academy of Agricultural Sciences/Key Lab of Plant Nutrition and Nutrient Cycling, Ministry of Agriculture, Beijing 100081, China; 2 Southwest University, Chong Qing 400715, China; 3 Institute of Plant Nutrient and Environmental Resources, Henan Academy of Agricultural Sciences, Zhengzhou 450002, China; Pacific Northwest National Laboratory, UNITED STATES

## Abstract

Biochar (BC) addition to soil is a proposed strategy to enhance soil fertility and crop productivity. However, there is limited knowledge regarding responses of soil respiration and C-cycle enzyme activities to BC and nitrogen (N) additions in a calcareous soil. A 56-day incubation experiment was conducted to investigate the combined effects of BC addition rates (0, 0.5, 1.0, 2.5 and 5.0% by mass) and urea (U) application on soil nutrients, soil respiration and C-cycle enzyme activities in a calcareous soil in the North China Plain. Our results showed soil pH values in both U-only and U plus BC treatments significantly decreased within the first 14 days and then stabilized, and CO_2_emission rate in all U plus BC soils decreased exponentially, while there was no significant difference in the contents of soil total organic carbon (TOC), dissolved organic carbon (DOC), total nitrogen (TN), and C/N ratio in each treatment over time. At each incubation time, soil pH, electrical conductivity (EC), TOC, TN, C/N ratio, DOC and cumulative CO_2_ emission significantly increased with increasing BC addition rate, while soil potential activities of the four hydrolytic enzymes increased first and then decreased with increasing BC addition rate, with the largest values in the U + 1.0%BC treatment. However, phenol oxidase activity in all U plus BC soils showed a decreasing trend with the increase of BC addition rate. Our results suggest that U plus BC application at a rate of 1% promotes increases in hydrolytic enzymes, does not highly increase C/N and C mineralization, and can improve in soil fertility.

## Introduction

Biochar (BC) is produced by the pyrolysis of biomass under high-temperature and oxygen-limited conditions [1]. It is a recalcitrant C-rich organic material, with a large surface area and highly aromatic structure [2]. Some studies have reported that BC has considerable potential to enhance soil quality and sequester carbon(C) due to its unique properties, such as increase in soil pH, electrical conductivity (EC), soil organic carbon (SOC), and soil holding capacity of nitrogen and other nutrient elements [3–4]. However, other studies have shown either no effect or a negative effect of BC on soil fertility parameters and C storage potential [5–6]. Mineral fertilization as an intensive management practice in the agricultural ecosystem could also affect soil C and N transformation processes [7]. Therefore, it is important to understand the variable effects of combined application of BC and mineral fertilization on soil properties.

Soil respiration, namely release of C in the form of CO_2_ in the process of SOC mineralization, is an important part of the soil source-sink relationship. BC can act as a sink or a source of C depending on the interactions between the BC and soil [8]. Previous studies have reported that a range of responses have been observed in regards to the priming effect of BC on SOC mineralization; positive [9–10], neutral [11–12] and negative [13]. These contradictory results are primarily attributed to the characteristics of BC and soil types and time durations used in the different studies [8, 14]. In addition, recent studies have shown that there is great uncertainty about the response of soil respiration to additions of BC and N fertilizer. Lu et al. [15] has reported that additions of BC and N fertilizer significantly decreased decomposition of native SOC and even inhibited the stimulation effect of N fertilization on the decomposition of native SOC. Conversely, Sui et al. [16] has found that additions of BC and N fertilizer enhanced CO_2_emission for relatively short periods. Accordingly, understanding the process of soil respiration and its key controlling factors is important for evaluating the ecosystem C budget.

Soil extracellular enzymes are the catalysts of organic matter decomposition and are involved in the biogeochemical cycling of nutrients [17]. Enzyme activities and their responses to added C and N have received considerable attention [18–19]. Some studies have reported that long-term N fertilization increases the activities of soil hydrolytic enzymes involved in labile C breakdown in conventional agricultural management practices [20–21], while other studies have shown that N fertilizer decreases the activities of some hydrolytic enzymes [22–23]. Similarly, available data reveal a variable effect of BCs on extracellular enzyme activities. Some studies have reported that BC addition to soil usually reduces the C-cycle enzyme activities [24–25], while other studies have found inconsistent results [26–27].The influence of BC on soil enzyme activity mainly depends on the interaction of substrate and enzyme with BC [26]. Sorption of substrates on BC CEC/AEC sites contributes to enzymatic reactions and further improves soil enzyme activities; however, binding of extracellular enzymes to the BC surface inhibits the enzymatic reaction [28]. However, to our knowledge, there is almost no information on the dynamic variation of the potential activities of C-cycle enzymes under the combined application of BC and N fertilizer.

The calcareous loamy soil in the North China Plain (NCP) is generally deficient in nutrients and SOM. At present, an intensive rate of N application is being used to meet the increasing demand for agricultural products in the region, which has led to low fertilizer use efficiency and serious environmental problems [29]. Thus, there is a need to find the appropriate soil management strategies to reduce N fertilization and enhance soil fertility. The meta-analysis of Steiner et al. [30] showed that the combination of BC and N fertilizer was effective for improving crop yield while reducing N fertilizer by 10%. However, there is little systematic research on potential mechanisms of C mineralization priming effects and C-cycle enzyme activities between BC and chemical N in a calcareous soil. Therefore, the specific objectives for this work were: 1) to investigate the short-term effects of different levels of BC application (0, 0.5, 1.0, 2.5 and 5.0% by mass) combined with U on the dynamic changes of soil pH, EC, TOC, TN, DOC and C/N ratio; and 2) to illustrate the changes of soilrespiration and potential activities of C-cycle enzymes after BC and U additions to a calcareous soil.

## Materials and Methods

Ethics statement: No specific permissions were required for a 56-day laboratory study, because soil samples are collected from Soil Fertility and Fertilizer Efficiency Monitoring Network Station, Zhengzhou, Henan Province, China, which is long-term formal cooperator. Furthermore, our study did not involve endangered or protected species.

### Soil selection and characterization

Soil was collected from the top layer (0–20 cm) of a calcareous soil with a light loamy texture at the Soil Fertility and Fertilizer Efficiency Monitoring Network Station, Zhengzhou, Henan Province, China (34°47′02″ N, 113°39′25″ E). The soil samples were air-dried and ground to pass through a 2-mm sieve. Some of the soil characteristics are shown in Table 1. Soil pH was measured with a compound electrode (PE-10, Sartorius, Germany) using a soil to water ratio of 1:2.5. Soil EC was determined in 1:5 (w/v; g cm^−3^) soil-water mixtures. The contents of TOC and TN in soil were determined using a total organic C/total N analyzer (Multi N/C 3100/HT1300, Analytik Jena AG, Germany). Inorganic N (NH_4_^+^-N and NO_3_^−^-N) was extracted with 2M KCl and subjected to flow injection analysis (TRAACA-2000, Germany).

**Table 1:** The physical and chemical properties of experimental soil and biochar.

	Yield (%)	pH	Ash content (%)	EC (mS cm^−1^)	Surface area (m^2^ g^−1^)	TOC (%)	TN (%)	NH_4_^+^-N (mg kg^−1^)	NO_3_^—^N (g kg^−1^)

biochar	32.60	10.50	22.28	5.37	4.00	53.81	1.22	/	/
soil		8.28	/	0.47	/	0.54	0.07	15.82	0.43

Abbreviations: TN, Total nitrogen, TOC Total organic carbon, EC Electrical conductivity. “/” not measured.

### Biochar characterization

The BC pyrolysis condition was as described by Wang et al. [31]. The BC was produced at 450°C by slow pyrolysis of maize straw (5°C min^−1^ heating and 1hresidence time in a Microwave Muffle Furnace (SX_2_, Shanghai Rongfeng Scientific Instrument Inc., Shanghai, China)). All BC samples were mixed evenly, ground and sieved to < 0.154 mm. Some of the BC characteristics are shown in Table 1. Yield was calculated according to the following equation: yield (%) = (weight of BC) / (weight of feedstock) ×100.The pH was measured by adding BC to deionized water at amass ratio of 1:20; the solution pH was measured with a pH meter (PP-20, Sartorius, Germany). To determine ash content, 1 g of the ground BC was heated at 600°C for 8 h in a muffle furnace and the ash (in percentage) was calculated from: Ash (%) = (weight of ash) / (weight of BC) × 100. Electrical conductivity (EC) was determined in 1:5 (w/v; g cm^−3^) BC-water mixtures. The BET surface area of BC was estimated as described by Dai et al. [32]. Elemental C and N contents were determined with an Elemental analyzer (varioPYRO cube, Elementar, Germany).

The functional group variability of BC was investigated by analysing Fourier transform infrared spectroscopy (VERTEX 70 FTIR, Bruker Corporation, Germany) using KBr pellet method in the scanned range of 4000–500 cm^−1^ with a resolution of 4 cm^−1^. The surface physical morphology of BC was examined using a scanning electron microscopy (SEM, FEI Quanta 200 FEG, America). BC Sample was analyzed in duplicate by energy spectrum analysis (EDS) to determine the relative elemental content.

### Incubation experiment

An incubation experiment was conducted over 56 days to estimate the effects of BC on soil nutrients, soilrespiration and potential activities of C-cycle enzymes. The six treatments were as follows: an untreated control (CK), urea (U) and U mixed with BC at rates of 0.5, 1.0, 2.5 and 5.0% by mass (hereafter termed U+0.5%BC, U+1.0%BC, U+2.5%BC and U+5.0%BC, respectively). Specifically, mixing 150g of 2-mm sieved soil with the appropriate quantity of BC amendment was placed in 500-mL plastic containers. A urea solution was added to each container (except CK) at the rate of 200 mg N (kg soil)^−1^. Deionized water was added to each soil mixture to maintain 40–45% of the water-holding capacity. Each container was sealed with a polyethylene film containing 3 pin-sized holes to permit aeration. All of the soil treatments were incubated at 25°C. Each of the treatments was replicated 18 times. The Soil from three replicates was destructively sampled to determine the pH, EC, TOC, DOC, and TN at 1, 3, 7, 14, 28 and 56 days of incubation, and C-cycle enzyme activities at days 7, 14, 28 and 56.The DOC and total dissolved nitrogen (TDN) were extracted with 0.01M CaCl_2_and determined by a total organic C/N analyzer (Multi N/C 3100/HT1300, Analytik Jena AG, Germany).

The CO_2_emission rate was measured by trapping CO_2_from the container headspace in small vials containing 1 M NaOH solution. The containers were hermetically sealed with a polyethylene film, and incubated in the dark for 56 days at 25°C. The NaOH solution in the vials was replaced after 1, 3, 7, 14, 21, 28, 35, and 42 days. The CO_2_ evolved during the incubation was trapped in 1 M NaOH, the excess of which was then titrated with 0.1 M HCl after addition of BaCl_2_. Mineralized C was calculated as the CO_2_emission rate (μg g^−1^soil h^−1^) and cumulative CO_2_emissions (g kg^−1^ soil) according to El-Naggar et al. [4].

In order to calculate the direct effect of BC addition on CO2-C evolved from soil, the average value of CO_2_-C measured at each incubation time from the U-only treatment was subtracted from each BC plus U treatment to obtain the cumulative values, which were fitted to different kinetics functions. The best fits were obtained by first-order kinetic equation: Cmin = C_0_ (1-e^−Kt^), being Cmin the C mineralized, C_0_ the potentially mineralizable pools of organic C, K the mineralization rate constant, and t the time. The index C_0_*K/added C is an indicator of degradability for organic materials. The coefficient of Akaike Information Criterion (AIC) was used as an important criterion of regression models to decide the best fit, which calculated according to the following equation: AIC = n·ln(RSS)+2(P+1)-n·ln(n), being RSS residual sum of squares, n sample size, and P the number of independent variables in regression equations. The fits and kinetic parameters were carried out using the SigmaPlot 12.5 software.

### Enzyme activity

In this study, we analyzed the following C-cycle enzymes: four different hydrolytic enzymes (β-D-cellobiosidase, β-glucosidase, β-xylosidase and α-glucosidase) and phenol oxidase. The potential activities of hydrolytic enzymes were quantified according to fluorescence-based protocols as described in Ai et al. [33]. Briefly, 1 g fresh soil was homogenized in 100-mL sterilized water using a polytron homogenizer, and then a magnetic stirrer was used to maintain a uniform suspension. The sterilized water, sample suspension, 10 μM references, and 200 μM 4-methylumbelliferyl (MUB)-linked substrates were dispensed into the wells of a black 96-well microplate. The microplates were covered and incubated in the dark at 25°C for 4 h. After incubation, 10 μL of a 1-M NaOH solution was rapidly added to each well of the microplate to stop the enzyme reaction. Fluorescence was quantified using a microplate fluorometer (Scientific Fluoroskan Ascent FL, Thermo, USA) with 365 nm excitation and 450 nm emission filters. The activities of hydrolytic enzymes were expressed in units of nmol h^−1^ g^−1^. The non-fluorometric enzyme (phenol oxidase) was measured spectrophotometrically in the clear 96-well microplate using the substrate of _L_-3,4-dihydroxyphenylalanine (L-DOPA).The sterilized water, sample suspension, and 25mM L-DOPA were the same as for the fluorometric enzymes [33]. Phenol oxidase activity required a 20-h incubation time to cause significant color change, which was quantified using a microplate spectrophotometer with 450 nm absorbance and expressed in units of μmol h^−1^ g^−1^. The activities of enzymes were calculated according to DeForest et al. [34].

### Statistical analysis

All statistical analyses were performed using the software package SAS version 8.0 (SAS Institute, Inc., Cary, NC, USA). The data were statistically assessed through two-way ANOVA between means within the factors (treatments and days of incubation). The means and standard deviations (±SD) of three replications were reported. Comparisons of means were conducted using the LSD test, at *P* = 0.05. Redundancy analysis (RDA) with the Monte Carlo permutation test (499 permutations) was performed to determine if soil enzyme activities were correlated with variation of soil physic-chemical parameters, using the software Canoco version 5.0 (Microcomputer Power Inc., Ithaca, NY, USA).

## Results

### Biochar characteristics

**Fig 1:**
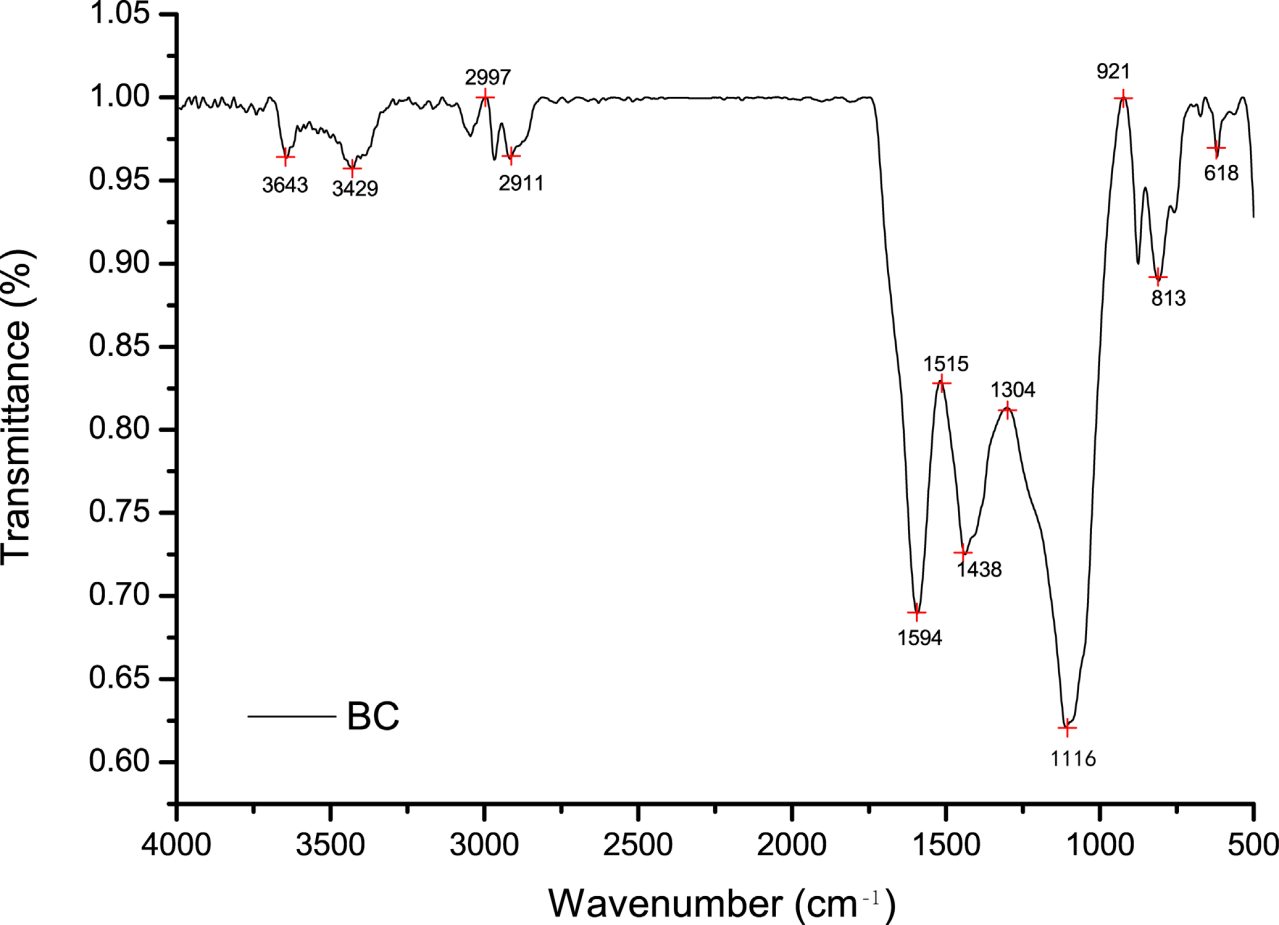
FTIR spectra of maize straw biochar at 450°C.

The FTIR spectra of BC sample was in the range of 4000−500 cm^−1^ (Fig 1). It could be seen that the relatively weak peaks at 3429 and 3643 cm^−1^weredue to O-H stretching vibration absorption of amino and hydroxyl groups. A smaller band from 2800 to 3000 cm^−1^ (peak at 2911 cm^−1^) was likely due to alkyl C-H stretches. A sharp spectral peak at1594 cm^−1^ was associated with aromatic C = O stretching. The peak at1438 cm^−1^ could be associated with aromatic rings and could be associated to phenolic groups. The intense band observed at 1111 cm^−1^wasascribed to the C–O stretching vibration of the alcoholic groups. A sharp spectral peak at 813 cm^−1^ was assigned to aromatic C-H stretching. The weak peak at about618 cm^−1^ could be assigned to C–H out-of-plane bending in aromatic derivatives. This result showed that material was mostly carbonaceous with a matrix of highly cross-linked network.

**Fig 2:**
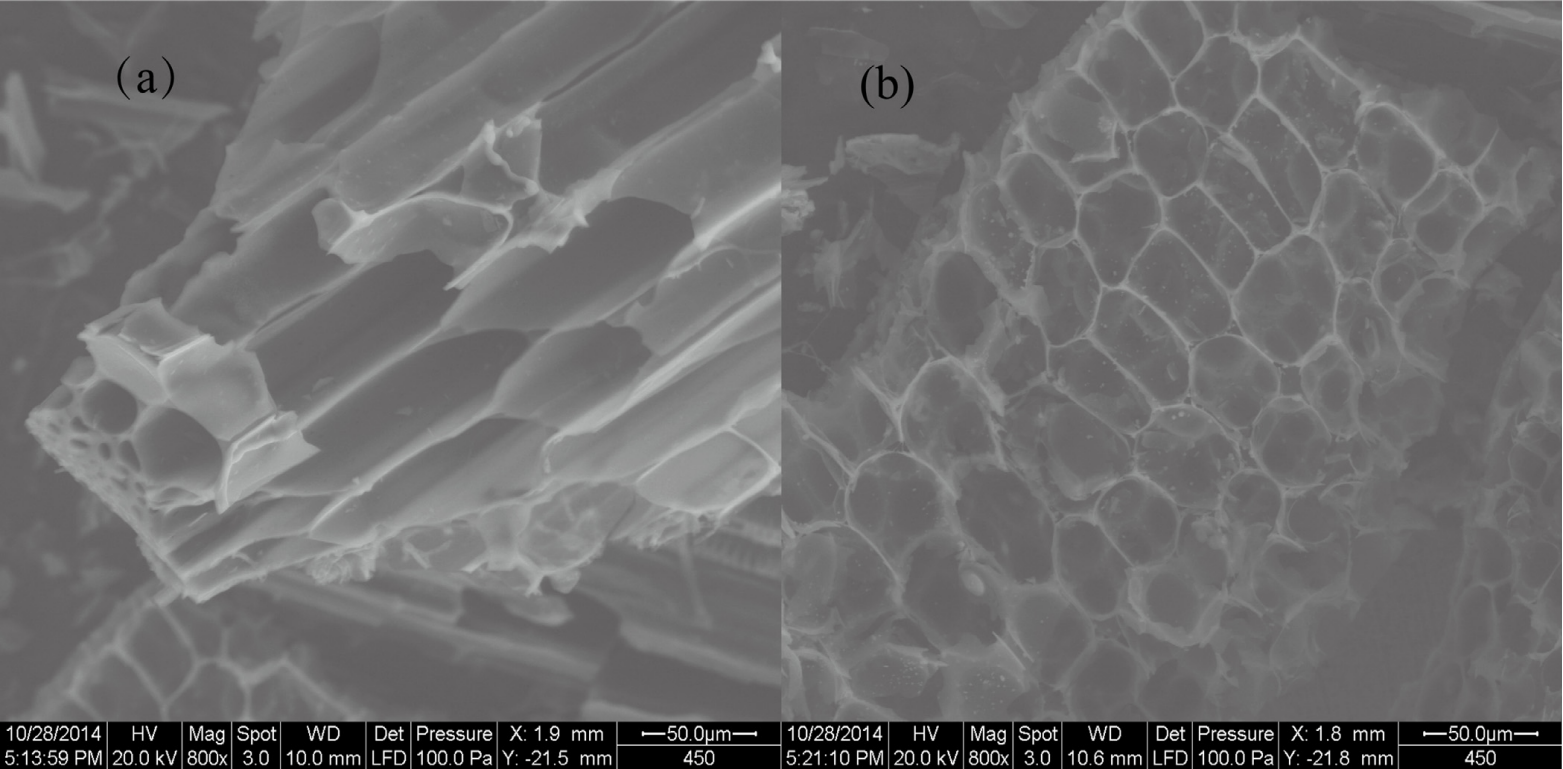
Scanning electron microscope (SEM) images of maize biochar produced at 450°C (a, b). (a) denotes the cross section of biochar, (b) denotes longitudinal section of biochar.

**Fig 3:**
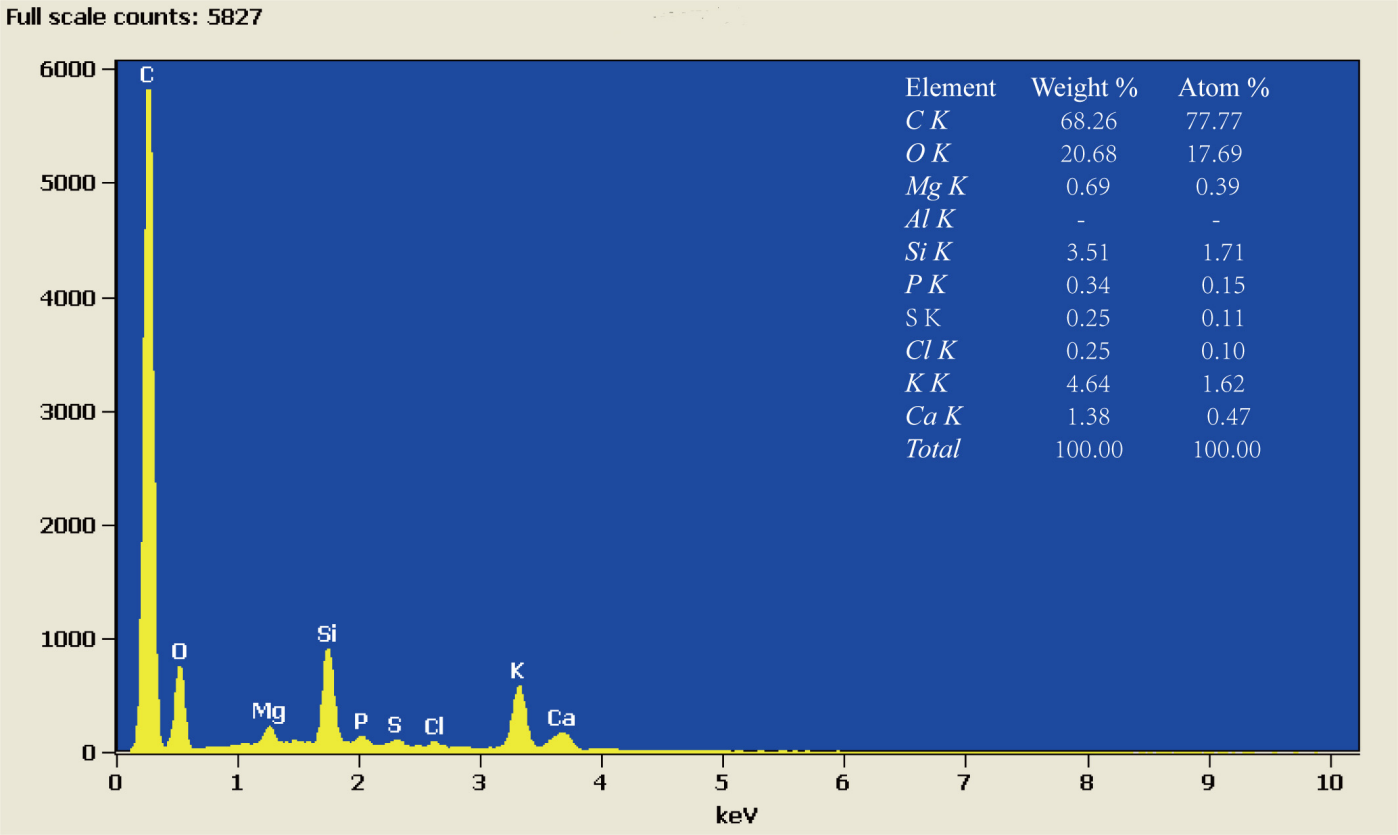
Energy spectrum analysis for biochar surface produced at 450°C.

The SEM images were used to visually display the variation in pore surface structure of BC produced at 450°C. This result showed that the cross section of BC sample had an obvious tubular pore structure, with most of the pores adhering to some particular substances in their walls (Fig 2A). The outer surface profile of BC sample showed a typical aligned and clear rectangular structure, with thin cell walls adhered to some particles (Fig 2B). The elemental composition of BC was dominated by C (68.26% Weight), followed by O (20.68% Weight) and then minor mineral elements (e.g. Ca, Mg, Si, P, K, etc.) (Fig 3). Based on this observation, it can be concluded that BC had a porous morphology, and relatively rich in nutritional and mineral elements.

### Dynamics of pH and EC in soil

**Fig 4:**
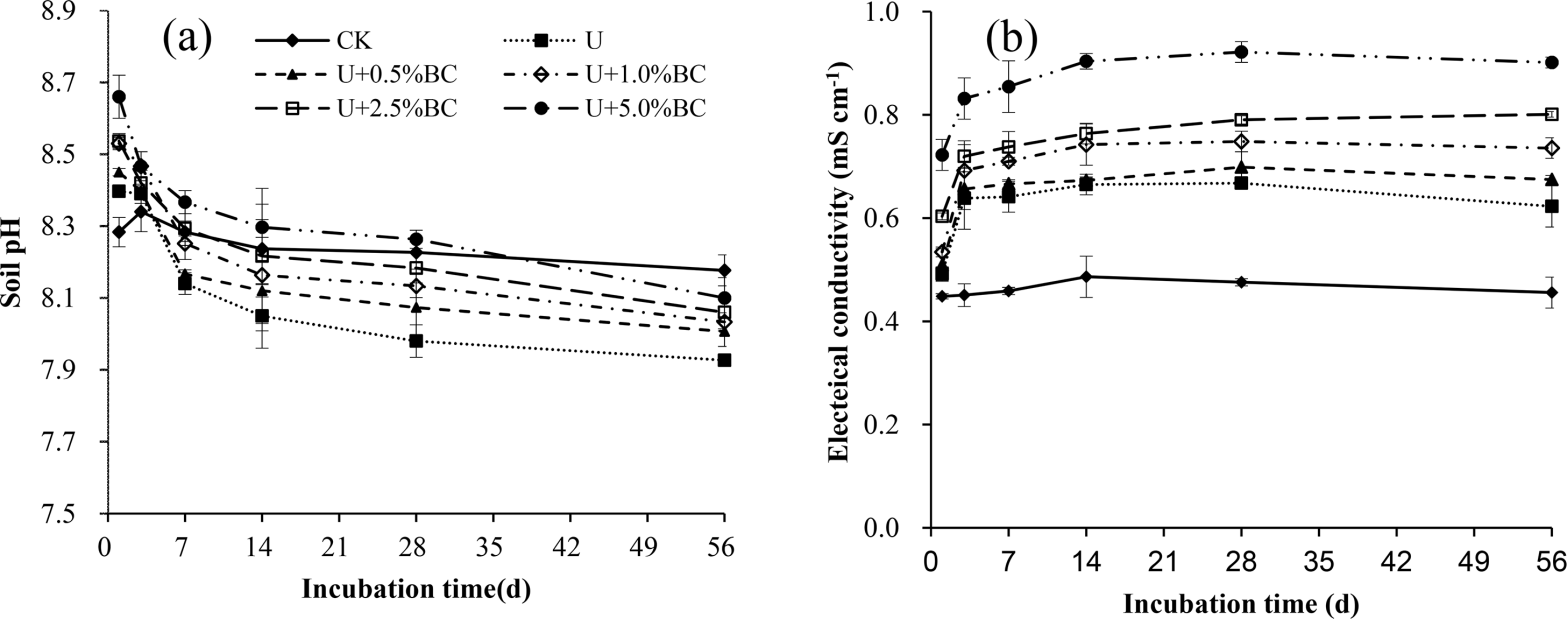
Dynamic variation of soil pH (a) and electrical conductivity (b) in different treatments over a 56-day incubation. Vertical bars in the figures represent standard error of the means (*n* = 3).

Soil pH changed little for the CK treatment over time, and significantly decreased for both U-only and U plus BC treatments within the first 14 days (*P*< 0.05), and then stabilized (Fig 4A). Soil pH values in both U-only and U plus BC treatments were significantly higher than those of CK on Days 1 and 3, with an average increase of 0.23 and 0.09 units, respectively. However, pH values on Day 56 for both U-only and U plus BC soils were significantly lower than that of CK by 0.08–0.25 units (*P*< 0.05). We also found that pH values in all U plus BC soils increased with the increase of BC addition rate at each incubation time, with the U+5%BC treatment showing an average of about 0.21 units higher than the U-only treatment.

In this study, Soil EC in the CK treatment did not vary over time, while the EC in both U-only and U plus BC soils increased significantly from Day 1 to 3 (*P*< 0.05), and did not vary from Day 7 to 56 (Fig 4B). At each incubation time, the EC in both U-only and U plus BC soils increased significantly compared with CK, with the increases of 50.00–85.03% (*P*< 0.05). In all U plus BC soils, higher EC values were observed as BC application rates increased (Fig 4B).

### Dynamics of TOC, DOC, TN and C/N

**Fig 5:**
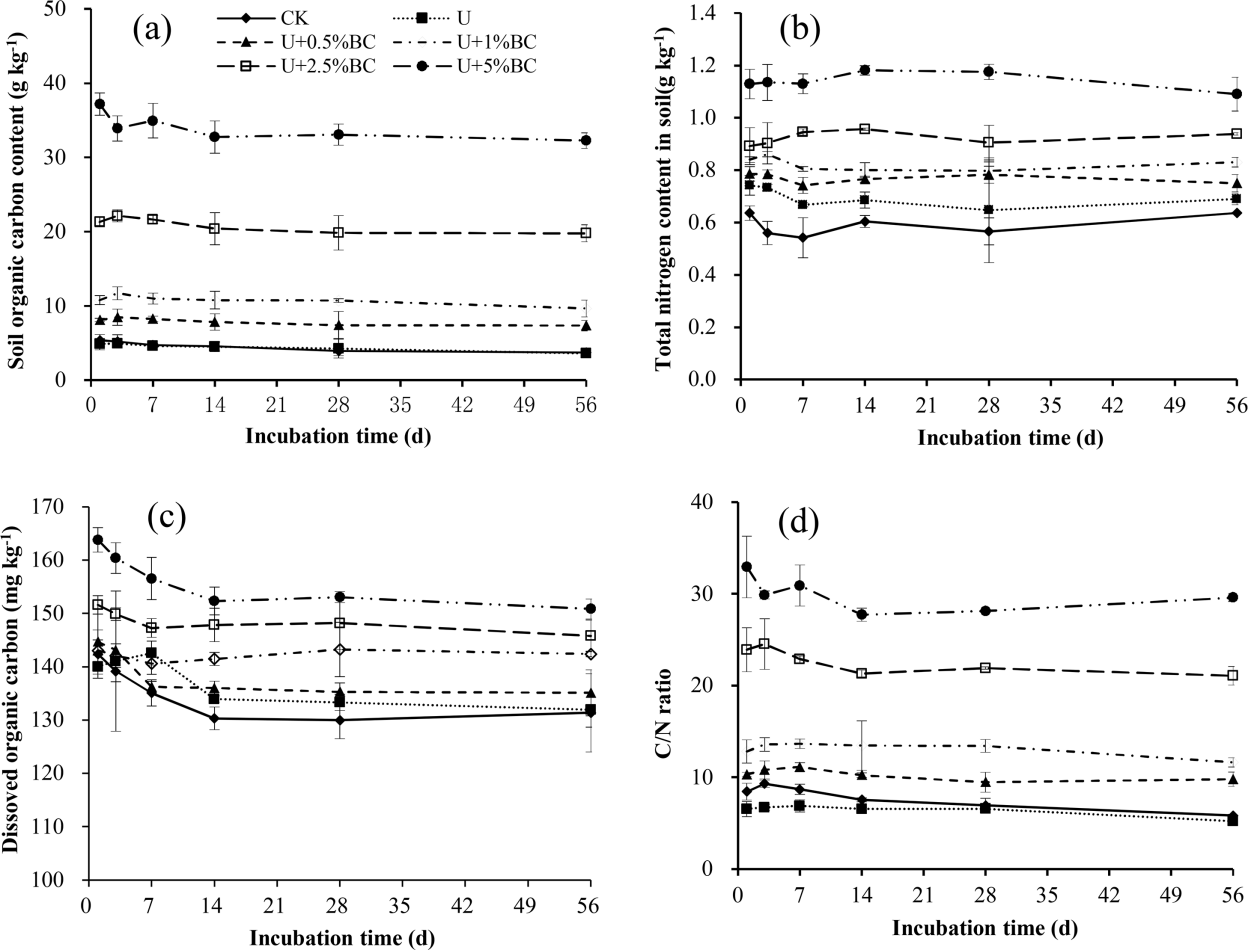
Dynamic variation of the contents of soil organic carbon (a), soil total nitrogen (b), dissolved organic carbon (c), and (d) C/N ratio in different treatments over a 56-day incubation. Vertical bars in the figures represent standard error of the means (*n* = 3).

The soil TOC content in each treatment gradually decreased over time (Fig 5A), but there was no significant difference (*P*> 0.05). At each incubation time, soil TOC contents in all U plus BC treatments were significantly higher than those of the CK and U-only treatments (*P*< 0.05), but there was no significant difference between the CK and U-only treatments (Fig 5A). The TOC content in soil significantly increased with increasing BC addition rate (*P*< 0.05), with the U+5%BC treatment showing an average of about6.42 and 6.62 times higher than the CK and U-only treatments, respectively(Fig 5A). The soil TN content for each treatment had no significant difference over time (Fig 5B). At each incubation time, the soil TN content in the U-only treatment significantly increased compared with CK (*P*< 0.05), with an average increase of 17.36%. The TN content at each incubation time significantly increased with the increase of BC addition rate (Fig 5B). The TN contents in all U plus BC soils were significantly higher than the U-only treatment, with an average increase of 9.10–62.46%.

The DOC contents in each treatment showed a decreasing trend with over time (Fig 5C). At each incubation time, the DOC content in soil increased with increasing BC addition rate, with the U+5%BC treatment showing an average of about 15.93 and 12.63%higher than the CK and U-only treatments, respectively (Fig 5C).

There was no significant difference in soil C/N ratio for each treatment over time (*P*> 0.05). The lowest C/N ratio was found in the U-only treatment at each incubation time (Fig 5D). In addition, The C/N ratio in all U plus BC soils significantly increased with increasing BC addition rate (*P*<0.05), which was an average of 2.83 and 3.64 times higher in the U+5%BC treatment than in the CK and U-only treatments, respectively (Fig 5D).

### Soil respiration and carbon mineralization kinetics

**Fig 6:**
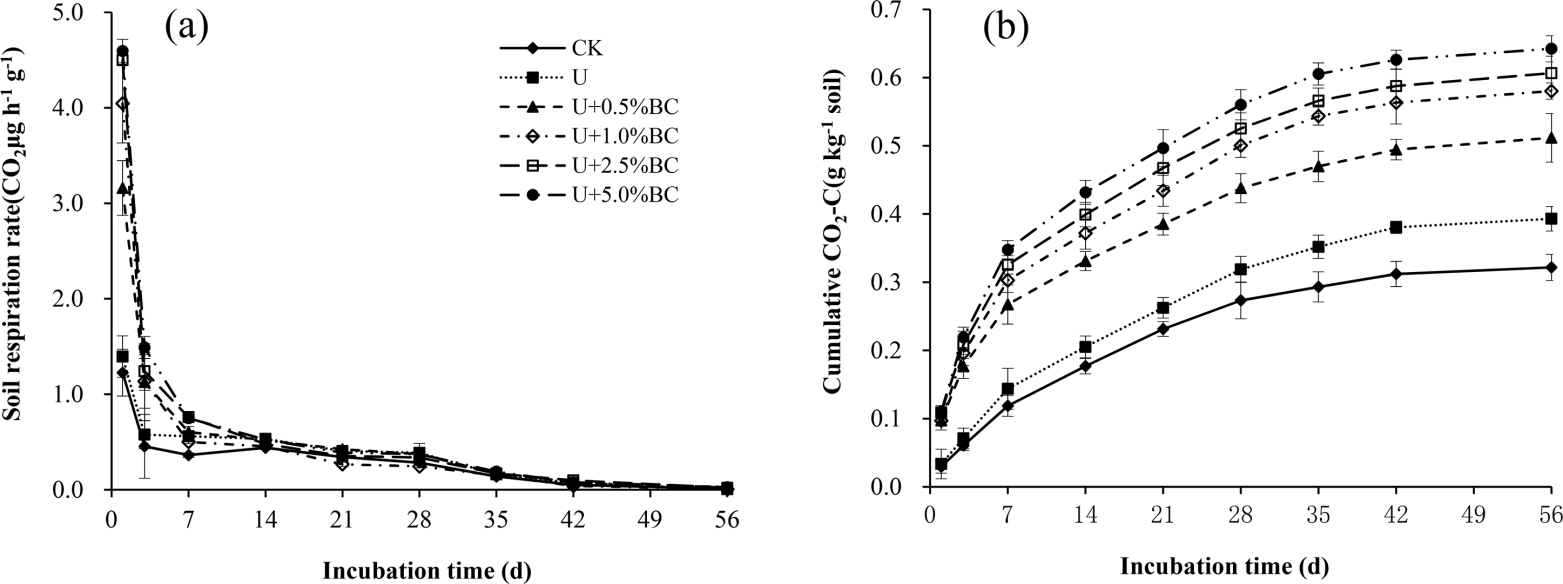
Response of soil respiration to additions of biochar and urea during a 56-day incubation: soil CO_2_ emission rate (a), cumulative CO_2_ emissions (b).Vertical bars in the figures represent standard error of the means (*n* = 3).

Throughout the monitoring period, the CO_2_ emission rate in all U plus BC soils decreased exponentially over time; the largest values were found on Day 1,and decreased<0.60CO_2_μg h^−1^ g^−1^ after 7 days, similar to the level of the CK treatment(Fig 6A).In the first 7 days of incubation, the soil CO_2_emission rate in the U-only treatment increased slightly compared with CK. TheCO_2_ emission rate increased with the increase of BC addition rate, and the U+5.0%BC treatment was 2.75 and 2.29times higher than the CK treatment at Days 1 and 3, respectively (Fig 6A). However, CO_2_emission rate had no significant difference between all treatments at the later stages of incubation (*P*> 0.05). In this study, the cumulativeCO_2_ emission in the U-only treatment was significantly higher than in the CK treatment (Fig 6B). The cumulative CO_2_ emissions in all U plus BC soils were significantly higher than those of the CK and U-only treatments (*P*<0.05), with increases of 22.2–99.7% and 30.2–63.4%, respectively (Fig 6B). Soil cumulative CO_2_ emissions increased with increasing BC addition rate, with the maximum value found in the U+5.0%BC treatment (0.64gCO_2_ kg^−1^).

The direct effect of BC addition on CO_2_-C evolved from soil was fitted to a first-order kinetic model Cmin = C_0_ [1 − e^−Kt^], with the smaller AIC values (Table 2). The potentially mineralizable pool of organic C (C_0_) in all U plus BC soils increased with increasing BC addition rate, but K and index C_0_*K/added C showed the same decreasing trend.

**Table 2:** Parameter of first-order kinetic model (Cmin = C_0_ [1-e^-Kt^]) fitted to the cumulative values of CO_2_-C mineralized from different amounts of BC application.

Parameter	U+0.5%BC	U+1.0%BC	U+2.5%BC	U+5.0%BC
Add C(g kg^-1^)	2.691	5.381	13.453	26.905
C_0_ (g kg^-1)^	0.121	0.179	0.207	0.241
K (d^-1^)	0.730	0.402	0.379	0.322
% C remaining ^a^	95.54	96.67	98.47	99.10
C_0_*K/add C	0.033	0.013	0.006	0.003
AIC	28.26	33.31	34.81	36.08
*F*-value	186.969**	170.117**	257.61**	290.138**

^a^ Proportion of amendment C remaining in soil at the end of incubation.

** = significant with *P*<0.001

### Dynamics of soil C-cycle enzyme activities

**Fig 7:**
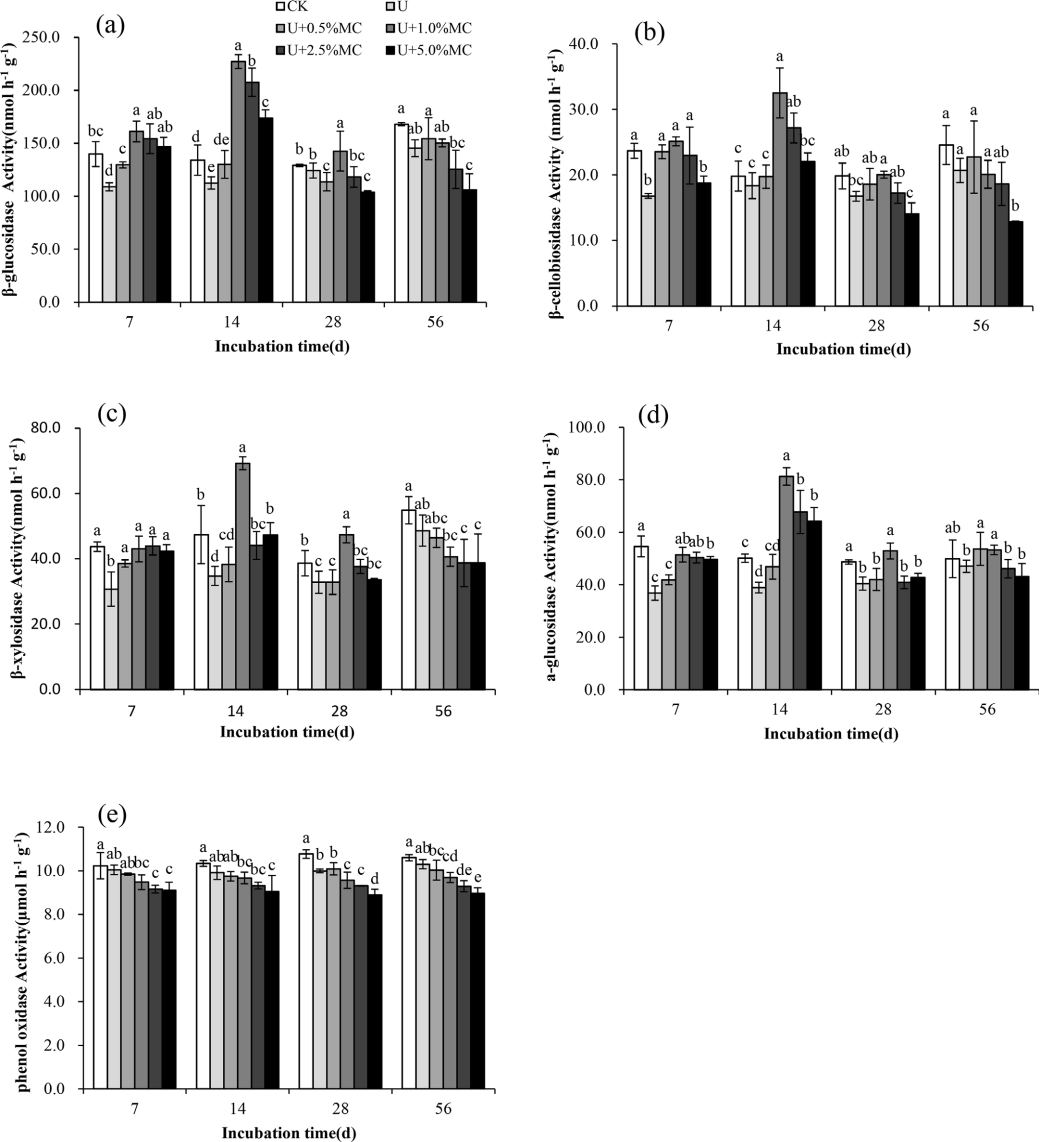
Response of C-cycle enzyme activities to additions of biochar and urea during a 56-day incubation. (a) β-glucosidase activity, (b) β-cellobiosidase activity, (c) β-xylosidase activity, (d) α-glucosidase activity and (e) phenol oxidase activity. Vertical bars represent the standard error of the means (*n* = 3) and lower case letters indicate significant differences between treatments at the *P*< 0.05 level.

In the U+1.0%BC, U+2.5%BC, and U+5.0%BC treatments, the potential activities of the four hydrolytic enzymes (β-glucosidase, β-cellobiosidase, β-xylosidase and α-glucosidase) significantly increased, decreased, and then stabilized over time (*P*< 0.05); the peak enzyme activities occurred on Day 14 (Fig 7A–7D). For the CK, U-only and U+0.5%BC treatments, the potential activities of the four hydrolytic enzymes were stable in the first 28 days, then slightly increased after 28 days of incubation.

At each incubation time, the potential activities of the four hydrolytic enzymes in the U-only treatment were lower than the CK treatment, with decreases of 3.80–22.08, 7.35–29.08, 11.31–29.68 and 8.78–32.45%, respectively (Fig 7A–7D).The activities of the four enzymes changed differently in each U plus BC treatment. The activities of the four hydrolytic enzymes increased and then decreased with increasing BC addition rate on Days 7, 14 and 28, with the largest activities in the U+1.0%BC treatment. However, the activities of the four hydrolytic enzymes in all U plus BC soils significantly decreased with increasing BC addition rate on Day 56 of incubation (*P*<0.05).

The activity of phenol oxidase had no significant difference for each treatment over time (Fig 7E) (*P*< 0.05). At each incubation time, the largest value of phenol oxidase activity was observed in the CK treatment. The phenol oxidase activity in soil (except on day 28) had no significant difference between the CK and U-only treatment (*P*>0.05). The phenol oxidase activity in all U plus BC soils showed a decreasing trend with increasing BC addition rate at each incubation time, with the U+5%BC treatment showing significantly lower than the CK and U-only treatments by an average of about 14.08 and 10.50% (*P*< 0.05).

**Fig 8:**
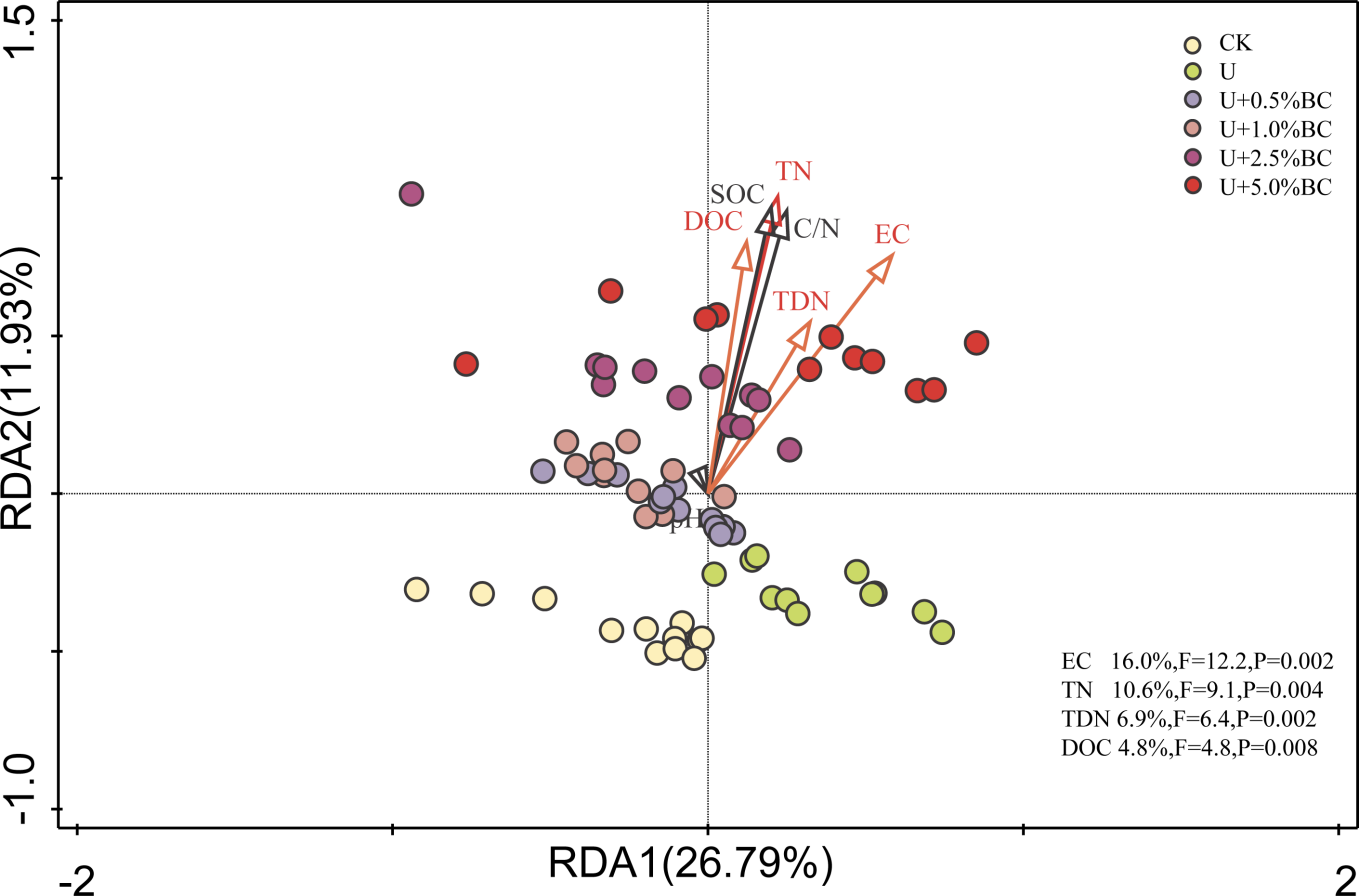
Redundancy analyses (RDA) of the correlations between soil parameters and enzyme activity. The red arrows indicate the soil parameters that had a significant impact on enzyme activities (P < 0.05), and the corresponding explained proportion of variability is shown in the lower right corner. Abbreviations: TN, total nitrogen; TOC, total organic carbon; DOC, dissolved organic carbon; TDN, total dissolved nitrogen and EC, electrical conductivity.

In addition, the redundancy analysis (RDA) showed that EC (F = 12.2, *P* = 0.002), TN (F = 9.1, *P* = 0.004), TDN (F = 6.4, *P* = 0.002) and DOC (F = 4.8, *P* = 0.008) were significantly correlated with soil enzyme activities and explained 16.0, 10.6, 6.9 and 4.8% of the total enzyme activity variability, respectively (Fig 8).

## Discussion

### Effects of BC and U additions on soil pH and EC

Soil pH, an important factor for reflecting soil properties, greatly influences soil fertility and thus plant growth. Some studies have reported that manure has less effect on soil pH when soil pH values are approaching neutrality, but mineral fertilizer containing a large proportion of urea can lead to decreases in soil pH [35–36]. Song et al. [37] also found that both ammonium nitrate and urea additions significantly decreased soil pH at different incubation periods compared with CK. Our results indicated that the pH values in both U-only and U plus BC soils initially increased and then decreased compared with CK over time. The transient increase in soil pH may be due to the NH_4_^+^ produced through U hydrolysis, after which NH_4_^+^ is turned into NO_3_-N, along with the release of H^+^ by nitrification, which is an important reaction after addition of N fertilizer into soil [38]. Once added to soil, abiotic and biotic surface oxidation of BC results in increased surface carboxyl groups, a greater negative charge, and subsequently an increasing ability to sorb cations [39]. Our results indicated that pH values in all U plus BC soils increased with the increase of BC addition rate because of many mineral elements (Ca, Mg, Si, K, etc.) contained in BC produced at 450°C (Fig 3). This is similar to the results of Laird et al. [40], who found significant increases in pH values with the amount of BC added in a typical Midwestern agricultural soil. The key reason for these increases of soil pH after BC addition can reduce the level of exchangeable H^+^ and Al^3+^by BC sorption [41].

Soil EC, a measure of soil water-soluble salt, can be used for identifying whether the content of soil water-soluble salt limits crop growth. Our results showed that the soil EC in both U-only and U plus BC treatments significantly increased from Day 1 to 3, and then tended to be stable (Fig 4B). Significant increase in EC could be due to the sudden presence of high concentration of soluble salts [3]. Soil EC has been reported to change after BC addition to soil [3, 42].Our results showed that EC values in all U plus BC soils significantly increased with increasing BC addition rate(Fig 4B). This is similar to the findings of Smider et al. [42], who reported that higher EC values were observed as BC addition rates increased. The results suggested that soil EC significantly increased with BC application because of the higher content of water soluble cations (K, Ca, Na, Mg, etc.) released from BC [31, 43].

### Effects of BC and U additions on TOC, DOC, TN and C/N

BC is widely proposed as a means to enhance soil quality and sequestering carbon(C), which is attributed to changes in soil physicochemical properties and biological functions [4, 44]. Our results indicated that the contents of TOC and TN in all U plus BC soils significantly increased with increasing BC addition rate, in agreement with the results of Sui et al. [16] and Smider et al. [42], suggesting that the BC may be useful for building C and N contents in soil, possibly because BC produced by slow pyrolysis process at low temperature (450°C) contains higher contents of C and N that can be measured in the mixture [45]. Soil C/N ratio is a critical factor influencing C and N transformation process [46]. Our results showed that the soil C/N ratio increased with increasing BC addition rate (Fig 5D), in agreement with the results of Sui et al [16], who found that soil C/N ratio in BC addition alone or BC plus N treatment was significantly higher than the CK, being ascribed to BC produced at 450°Cwith high initial C/N ratio (Table 1). There was no significant difference in soil C/N ratio for each treatment over time, indicating a slow transformation and recycling of organic matter [47], which is largely attributed to BC’s chemical recalcitrance [48].

Although DOC represents only a small proportion of soil organic carbon, it remains important in the soil ecosystem because of its mobility and reactivity [49]. Some studies have reported that BC addition increased soil DOC in the short-term [50–51]. However, Jones et al. [52] observed that BC addition to soil had less or no effect on DOC in a three-year study. Our results showed that DOC content at each incubation time increased with the increase of BC addition rate (Fig 5C), which was also reported by El-Mahrouky et al. [51], who found that BC addition to soil resulted in the short-term significant increases in soil DOC. This increase may be due to the fact that BC contains labile C and could release organic C into the soil [45].

### Soil respiration response to additions of BC and U

Our results indicated that U-only addition stimulated the decomposition of native organic C compared with CK, with 5.4 g organic C kg^−1^ in the calcareous soil of NCP. This finding is in agreement with results reported by Garland et al. [53], but different from those of Ni et al. [54] who observed a significant suppression effect by N amendment in the black soil of Northeast China, with 16.2 g organic C kg^−1^. The divergent results suggested that the effects of N fertilization on soil respiration mainly depended on the concentration of easily decomposed soil organic C [55]. Our results also found that all U plus BC treatments significantly increased cumulative CO_2_ emissions compared with the U-only treatment, showing an increased trend with increasing BC addition rate. This is similar to the results of Sui et al. [16], who found that additions of BC and N fertilizer enhanced CO_2_ emission in a relatively short-term. Priming effects induced by BC may be caused by its content of dissolved or volatile organic C [56]. Previous studies have confirmed that BC produced at low temperatures can stimulate C mineralization due to decomposition of labile components of BC over a short-term [57]. In addition, our results showed the direct effect of BC addition on CO2-C evolved from soil was fitted to a first-order kinetic model Cmin = C_0_ [1 − e^−Kt^], which is possibly due to dissociation of carbonates and other labile C fraction during the early stages of incubation [58–59]. This was further correlated with carbonate dissociation by biotic and abiotic factors in early phase of BC applied soil [58]. Conversely, Prayogo et al. [14] reported that increasing BC application to soil from 1 to 30% w/w caused progressively greater reduction of CO_2_.Potential mechanisms of negative priming effects may be ascribed to the sorption of labile C onto the surface or into the pore network of BC [25].

### Responses of the soil C-cycle enzyme activities to additions of BC and U

Extracellular enzymes are an important factor for driving the C/N transformation in soil, and the potential enzyme activities have been used for decades as indicators of soil quality and nutrient cycling [22]. Helfrich et al. [60] has reported that soil C/N ratio can change the microbial population and activity. Our results showed that the potential activities of the four hydrolytic enzymes were lower in the U-only treatment than the CK treatment (Fig 7A−7D), which is similar to the results of Zhou et al. [22], who reported that N fertilizer decreased the C-related hydrolytic enzyme activities. The possible explanation for the decreased enzyme activities was a lower soil C/N ratio (an average of 6.43) after U-only addition. After BC addition to soil, responses of the hydrolytic enzyme activities to BC become more complex. The increased enzyme activities could be related to the microbial availability of a higher quantity of substrates within lower BC-amended soils during the early stages of incubation [61].Conversely, the decreased enzyme activities were most likely due to the sorption of enzymes by BC and subsequent masking of active sites, rather than sorption of substrates or products, presumably caused by excessive BC porosity and a reactive surface area [28]. Previous studies have stated that there is great uncertainty about impacts of BC addition on the activities of hydrolytic enzymes involved in C cycling [24–27]. Our results showed that the activities of the four enzymes in all U plus BC soils increased, then decreased with increasing BC addition rate at days7, 14 and 28 of incubation, but significantly decreased at day 56 of incubation (Fig 7A−7D). In addition, our results also founded that the contents of soil EC, TN, TDN and DOC were significantly correlated with soil enzyme activities. The divergent results indicated that responses of the hydrolytic enzyme activities to BC may primarily depend on the amount of BC addition, soil nutrient content and time since BC application [14, 57].

Soil phenol oxidase is an enzyme excreted mainly by microorganisms, which catalyzes the oxidation of recalcitrant aromatic compounds, such as lignin, into more readily available substrates using oxygen as the final electron acceptor [62]. Low phenol oxidase activity contributes to the accumulation of soluble phenolics and inhibits the activities of hydrolytic enzymes, and thus benefits soil C sequestration [63–64]. We found that BC and U additions had no significant effect on phenol oxidase activity over time (*P* > 0.05).However, the phenol oxidase activity in all U plus BC soils showed a decreasing trend with the increase of BC addition rate at each incubation time (Fig 7E). This may be because BC addition slows the rate of substrate oxidation when there is high content of recalcitrant C in soil, and the response has been linked to reduction in phenol oxidase activity [18].

## Conclusions

This study clearly demonstrated the responses of soil nutrients, soilrespiration and C-cycle enzyme activities to BC and U additions within 56 days of incubation. Our results indicate that the change of soil pH was mainly attributed to the amounts of U and BC, while changes in soil EC, TOC, DOC TN and C/N ratio depended mainly on the BC addition rate and increased with the increase of BC addition rate. The BC addition induced a rapid increase in C mineralization at the early stages of incubation (i.e. within 7 days), and parameters of C mineralization kinetics suggested that there was the direct effect of BC addition rate on CO2-C evolved from soil in a short-term. The contents of soil EC, TN, DOC and TDN were dominant factors affecting soil enzyme activities. Our result indicated that the divergent variations in soil C-cycle enzyme activities may primarily depend on the amounts of U and BC additions, soil nutrient content and incubation time. Therefore, future studies, especially for long-term incubation and field plot, are needed to further estimate the effects of U and BC additions on the soil microbial activities involved in C cycling.

## Supporting Information

S1 File The Minimal Dataset. (XLSX)

## References

[1] IBI. Standardized product definition and product testing guidelines for biochar that is used in soil.2012. http://www.biochar-international.org/sites/default/files/guidelines_for_biochar_that_is_used_in_soil_final.pdf (cited 5 March, 2013).

[2] Kuzyakov Y, Bogomolova I, Glaser B. Biochar stability in soil: decomposition during eight years and transformation as assessed by compound-specific ^14^C analysis. Soil Biol Biochem. 2014; 70: 229–236.

[3] Masto RE, Ansari MA, George J, Selvi VA, Ram LC. Co-application of biochar and lignite fly ash on soil nutrients and biological parameters at different crop growth stages of *Zea mays*. Ecol Eng. 2013; 58: 314–322.

[4] El-Naggar AH, Usman ARA, Al-Omran A, Ok YS, Ahmad M, Al-Wabel MI. Carbon mineralization and nutrient availability in calcareous sandy soils amended with woody waste biochar. Chemosphere. 2015; 138: 67–73. 10.1016/j.chemosphere.2015.05.052 26037818

[5] Bruun EW, Ambus P, Egsgaard H, Hauggaard-Nielsen H. Effects of slow and fast pyrolysis biochar on soil C and N turnover dynamics. Soil Biol Biochem. 2012; 46: 73–79.

[6] Tammeorg P, Simojoki A, Mäkelä P, Frederick Stoddard LFL, Alakukku L, Helenius J. Short-term effects of biochar on soil properties and wheat yield formation with meat bone meal and inorganic fertiliser on a boreal loamy sand. Agr Ecosyst Environ. 2014; 191:108–116.

[7] Lee KH, Jose S. Soil respiration, fine root production, and microbial biomass in cottonwood and loblolly pine plantations along a nitrogen fertilization gradient. Forest Ecol Manag. 2003; 185: 263–273.

[8] Zimmerman AR, Gao B, Ahn MY. Positive and negative carbon mineralization priming effects among a variety of biochar-amended soils. Soil Biol Biochem. 2011; 43: 1169–1179.

[9] Bruun S, Clauson-Kaas S, Bobulska L, Thomsen IK. Carbon dioxide emissions from biochar in soil: role of clay, microorganisms and carbonates. Eur J Soil Sci. 2014; 65: 52–59.

[10] Cely P, Tarquis AM, Ferriero JP, Mendez A, Gasco G. Factors driving the carbon mineralization priming effect in a sandy loam soil amended with different types of biochar. Solid Earth. 2014; 5: 585–594.

[11] Mukherjee A, Lal R, Zimmerman A. Effects of biochar and other amendments on the physical properties and greenhouse gas emissions of an artificially degraded soil. Sci. Total Environ.2014; 487: 26–36. 10.1016/j.scitotenv.2014.03.141 24751592

[12] Zhang A, Liu Y, Pan G, Hussain Q, Li L, Zheng J, Zhang X. Effect of biochar amendment on maize yield and greenhouse gas emissions from a soil organic carbon poor calcareous loamy soil from Central China Plain. Plant Soil.2012; 351: 263–275.

[13] Keith A, Singh B, Dijkstra FA. Biochar reduces the rhizosphere priming effect on soil organic carbon. Soil Biol Biochem. 2015; 88: 372–379.

[14] Prayogo C, Jones JE, Baeyens J, Bending GD. Impact of biochar on mineralisation of C and N from soil and willow litter and its relationship with microbial community biomass and structure. Biol Fertil Soils. 2014; 50: 695–702.

[15] Lu W, Ding W, Zhang J, Li Y, Luo J, Bolan N, Xie Z. Biochar suppressed the decomposition of organic carbon in a cultivated sandy loam soil: A negative priming effect. Soil Biol Biochem. 2014; 76: 12–21.

[16] Sui YH, Gao JP, Liu CH, Zhang WZ, Lan Y, Li SH, Meng J, Xu ZJ, Tang L. Interactive effects of straw-derived biochar and N fertilization on soil C storage and rice productivity in rice paddies of Northeast China. Sci Total Environ. 2016; 544: 203–210. 10.1016/j.scitotenv.2015.11.079 26657366

[17] Burns RG, DeForest JL, Marxsen J, Sinsabaugh RL, Stromberger ME, Wallenstein MD, Weintraub MN, Zoppini A. Soil enzymes in a changing environment: current knowledge and future directions. Soil Biol Biochem. 2013; 58: 216–234.

[18] Grandy AS, Neff JC. Molecular C dynamics downstream: the biochemical decomposition sequence and its impact on soil organic matter structure and function. Sci Total Environ. 2008; 404: 297–307. 10.1016/j.scitotenv.2007.11.013 18190951

[19] Cusack DF, Silver WL, Torn MS, Burton SD, Firestone MK. Changes in microbial community characteristics and soil organic matter with nitrogen additions in two tropical forests. Ecology 2011; 92: 621–632. 21608471 10.1890/10-0459.1

[20] Bandick AK, Dick RP. Field management effects on soil enzyme activities. Soil Biol. Biochem. 1999; 31: 1471–1479

[21] Piotrowska A, Wilczewski E. Effects of catch crops cultivated for green manure and mineral nitrogen fertilization on soil enzyme activities and chemical properties. Geoderma 2012; 189–190: 72–80.

[22] Zhou X, Zhang Y, Downing A. Non-linear response of microbial activity across agradient of nitrogen addition to a soil from the Gurbantunggut Desert, northwestern China. Soil Biol Biochem. 2012; 47: 67–77.

[23] Wang RZ, Dorodnikov M, Yang S, Zhang YY, Filley TR, Turco RF, Zhang YG, Xu ZW, Li H, Jiang Y. Responses of enzymatic activities within soil aggregates to 9-year nitrogen and water addition in a semi-arid grassland. Soil Biol. Biochem. 2015a; 81: 159–167.

[24] Bailey VL, Fansler SJ, Smith JL, Bolton H. Reconciling apparent variability in effects of biochar amendment on soil enzyme activities by assay optimization. Soil Biol Biochem.2011; 43: 296–301.

[25] Lehmann J, Rillig MC, Thies J, Masiello CA, Hockaday WC, Crowley D. Biochar effects on soil biota-A review. Soil Biol Biochem. 2011; 43: 1812–1836.

[26] Lammirato G, Miltner A, Kaestner M. Effects of wood char and activated carbon on the hydrolysis of cellobiose by β-glucosidase from *Aspergillus niger*. Soil Biol Biochem. 2011; 43: 1936–1942.

[27] Paz-Ferreiro J, Fu S, Méndez A, Gasco G. Interactive effects of biochar and the earthworm Pontoscolex corethrurus on plant productivity and soil enzymes activities. J Soil Sediment. 2014; 14: 483–494.

[28] Czimczik CI, Masiello CA. Controls on black carbon storage in soils. Global Biogeochem Cy. 2007; 21(3): 1–11.

[29] Ju X, Xing G, Chen X, Zhang S, Zhang L. Reducing environmental risk by improving N management in intensive Chinese agricultural systems. P Natl Acad Sci USA. 2009; 106: 3041–3046. 10.1073/pnas.0813417106 PMC2644255 19223587

[30] Steiner C, Teixeira WG, Lehmann J, Nehls T, de Macedo JLV, Blum WEH, Zech W. Long term effects of manure, charcoal and mineral fertilization on crop production and fertility on a highly weathered central Amazonian upland soil. Plant Soil. 2007; 291: 275–290.

[31] Wang X, Song D, Liang G, Zhang Q, Ai C, Zhou W. Maize biochar addition rate influences soil enzyme activity and microbial community composition in a fluvo-aquic soil. Appl Soil Ecol. 2015b; 96:265–272

[32] Dai ZM, Meng J, Muhammad N, Liu XM, Wang HZ, He Y, Brookes PC, Xu JM. The potential feasibility for soil improvement, based on the properties of biochars pyrolyzed from different feedstocks. J. Soils Sediments 2013; 13: 989–1000.

[33] Ai C, Liang G, Sun J, He P, Tang S, Yang S, Zhou W, Wang X. The alleviation of acid soil stress in rice by inorganic or organic ameliorants is associated with changes in soil enzyme activity and microbial community composition. Biol Fertil Soils. 2015; 51(4): 465–477.

[34] DeForest JL. The influence of time, storage temperature, and substrate age on potential soil enzyme activity in acidic forest soils using MUB-linked substrates and L-DOPA. Soil Biol Biochem. 2009; 41: 1180–1186.

[35] Fan F, Li Z, Wakelin SA, Yu WT, Liang YC. Mineral fertilizer alters cellulolytic community structure and suppresses soil cellobiohydrolase activity in a long-term fertilization experiment. Soil Biol Biochem. 2012; 55: 70–77.

[36] Ge GF, Li ZJ, Fan FL, Chu GC, Hou ZN, Liang YC. Soil biological activity and their seasonal variations in response to long-term application of organic and inorganic fertilizers. Plant Soil, 2010; 326:31–44.

[37] Song YY, Song CC, Tao BX, Wang JY, Zhu XY, Wang XW. Short-term responses of soil enzyme activities and carbon mineralization to added nitrogen and litter in a freshwater marsh of Northeast China. Eur J Soil Biol. 2014; 61: 72–79.

[38] Grégory L, Roland P, Andrew N, Grünberger O, Chintachao W, Tessier D. Soil acidification without pH drop under intensive cropping systems in Northeast Thailand. Agric Ecosyst Environ. 2006; 114: 239–248.

[39] Cheng CH, Lehmann J, Engelhard M. Natural oxidation of black carbon in soils: changes in molecular form and surface charge along a climosequence. Geochim Cosmochim Ac. 2008; 72: 1598–1610.

[40] Laird DA, Fleming P, Davis DD, Horton R, Wang BQ, Karlen DL. Impact of biochar amendment on the quality of a typical Midwestern agricultural soil. Geoderma 2010; 158: 443–449.

[41] Van Zwieten L, Kimber S, Morris S, Chan KY, Downie A, Rust J, Joseph S, Cowie A. Effects of biochar from slow pyrolysis of paper mill waste on agronomic performance and soil fertility. Plant Soil. 2010; 327: 235–246.

[42] Smider B, Singh B. Agronomic performance of a high ash biochar in two contrasting soils. Agr Ecosyst Environ. 2014; 191: 99–107.

[43] Nguyen BT, Lehmann J. Black carbon decomposition under varying water regimes. Org Geochem. 2009; 40: 846–853.

[44] Biederman LA, Harpole S. Biochar and its effects on plant productivity and nutrient cycling: a meta-analysis. GCB Bioenergy. 2013; 5: 202–214.

[45] Ouyang L, Yu L, Zhang R. Effects of amendment of different biochars on soil carbon mineralisation and sequestration. Soil Res. 2014; 52:46–54.

[46] Wang J, Song C, Wang X, Song Y. Changes in labile soil organic carbon fractions in wet land ecosystems along a latitudinal gradient in North East China. Catena. 2012; 96: 83–89.

[47] Kindler R, Siemens J, Kaiser K, Walmsley DC, Bernhofer C, et al Dissolved carbon leaching from soil is a crucial component of the net ecosystem carbon balance. Glob Chang Biol. 2011; 17:1167–1185.

[48] McBeath AV, Ronald J. Smernik RJ. Variation in the degree of aromatic condensation of chars.Org Geochem. 2009; 40: 1161–1168

[49] Lin Y, Munroe P, Joseph S, Henderson R, Ziolkowski A. Water extractable organic carbon in untreated and chemical treated biochars. Chemosphere 2012; 87: 151–157. 10.1016/j.chemosphere.2011.12.007 22236590

[50] Beesley L, Dickinson N. Carbon and trace element fluxes in the pore water of an urban soil following greenwaste compost, woody and biochar amendments, inoculated with the earthworm *Lumbricus terrestris*. Soil Biol Biochem. 2011; 43: 188–196.

[51] El-Mahrouky M, El-Naggar AH, Usman AR, Al-Wabel M. Dynamics of CO_2_ emission and biochemical properties of a sandy calcareous soil amended with *Conocarpus* waste and biochar. Pedosphere 2015; 25(1): 46–56.

[52] Jones DL, Rousk J, Edwards-Jones G, DeLuca TH, Murphy DV. Biochar mediated changes in soil quality and plant growth in a three year field trial. Soil Biol Biochem. 2012; 45:113–124.

[53] Garland JL, Mackowiak CL, Zabaloy MC. Organic waste amendment effects on soil microbial activity in a corn-rye rotation: application of a new approach to community-level physiological profiling. Appl Soil Ecol.2010; 44: 262–269.

[54] Ni K, Ding WX, Cai ZC, Wang YF, Zhang XL, Zhou BK. Soil carbon dioxide emission from intensively cultivated black soil in Northeast China: nitrogen fertilization effect. J Soil Sediment.2012; 12: 1007–1018.

[55] Ding WX, Yu HY, Cai ZC, Han FX, Xu ZH. Responses of soil respiration to N fertilization in a loamy soil under maize cultivation. Geoderma 2010; 155:381–389.

[56] Spokas KA, Novak JM, Stewart CE, Cantrell KB, Uchimiya M, DuSaire MG, Ro KS. Qualitative analysis of volatile organic compounds on biochar. Chemosphere 2011b; 85: 869–882. 21788060 10.1016/j.chemosphere.2011.06.108

[57] Fang Y, Singh B, Singh BP. Effect of temperature on biochar priming effects and its stability in soils. Soil Biol Biochem. 2015; 80: 136–145.

[58] Bruun EW, Hauggaard-Nielsen H, Ibrahim N, Egsgaard H, Ambus P, Jensen PA, Dam-Johansen K. Influence of fast pyrolysis temperature on biochar labile fraction and short-term carbon loss in a loamy soil. Biomass Bioenerg. 2011; 35:1182–1189.

[59] Cross A, Sohi SP. The priming potential of biochar products in relation to labile carbon contents and soil organic matter status. Soil Biol Biochem. 2011; 43:2127–2134.

[60] Helfrich M, Ludwig B, Potthoff M, Flessa H. Effect of litter quality and soil fungi on macroaggregate dynamics and associated partitioning of litter carbon and nitrogen. Soil Biol Biochem. 2008; 40: 1823–1835.

[61] Tejada M, Garcia C, Gonzalez J, Hernandez M. Use of organic amendment as a strategy for saline soil remediation: influence on the physical, chemical and biological properties of soil. Soil Biol Biochem. 2006; 38: 1413–1421.

[62] Cullen D, Kersten PJ. Enzymology and molecular biology of lignin degradation In: Brambl R., Marzluf G.A. (Eds.), The Mycota III, Biochemistry and Molecular Biology. Springer-Verlag, Berlin, 1996; 295–306.

[63] Zibilske LM, Bradford JM. Oxygen effects on carbon, polyphenols, and nitrogen mineralization potential in soil. Soil Sci Soc Am J. 2007; 71:133–139.

[64] Sinsabaugh RL. Phenol oxidase, peroxidase and organic matter dynamics of soil. Soil Biol. Biochem. 2010; 42: 391–404.

